# Prolonged High Fat Diet Reduces Dopamine Reuptake without Altering DAT Gene Expression

**DOI:** 10.1371/journal.pone.0058251

**Published:** 2013-03-13

**Authors:** Jackson J. Cone, Elena H. Chartoff, David N. Potter, Stephanie R. Ebner, Mitchell F. Roitman

**Affiliations:** 1 Graduate Program in Neuroscience, University of Illinois at Chicago, Chicago, Illinois, United States of America; 2 Dept. of Psychiatry, Harvard Medical School, McLean Hospital, Belmont, Massachusetts, United States of America; 3 Department of Psychology, University of Illinois at Chicago, Chicago, Illinois, United States of America; Duke University Medical Center, United States of America

## Abstract

The development of diet-induced obesity (DIO) can potently alter multiple aspects of dopamine signaling, including dopamine transporter (DAT) expression and dopamine reuptake. However, the time-course of diet-induced changes in DAT expression and function and whether such changes are dependent upon the development of DIO remains unresolved. Here, we fed rats a high (HFD) or low (LFD) fat diet for 2 or 6 weeks. Following diet exposure, rats were anesthetized with urethane and striatal DAT function was assessed by electrically stimulating the dopamine cell bodies in the ventral tegmental area (VTA) and recording resultant changes in dopamine concentration in the ventral striatum using fast-scan cyclic voltammetry. We also quantified the effect of HFD on membrane associated DAT in striatal cell fractions from a separate group of rats following exposure to the same diet protocol. Notably, none of our treatment groups differed in body weight. We found a deficit in the rate of dopamine reuptake in HFD rats relative to LFD rats after 6 but not 2 weeks of diet exposure. Additionally, the increase in evoked dopamine following a pharmacological challenge of cocaine was significantly attenuated in HFD relative to LFD rats. Western blot analysis revealed that there was no effect of diet on total DAT protein. However, 6 weeks of HFD exposure significantly reduced the 50 kDa DAT isoform in a synaptosomal membrane-associated fraction, but not in a fraction associated with recycling endosomes. Our data provide further evidence for diet-induced alterations in dopamine reuptake independent of changes in DAT production and demonstrates that such changes can manifest without the development of DIO.

## Introduction

The overweight and obese represent an increasingly larger percentage of the United States and worldwide populations [1], [2]. While there are many pathways to obesity, perhaps one of the biggest threats to healthy body weight is the prevalence and consumption of highly palatable, densely caloric foods [3]. Indeed, the energy density (kcal/g) of food potently contributes to overweight and obesity in adults [4], [5]. Palatable foods evoke dopamine release in the striatum of both humans and non-human animals [6], [7], [8], [9] and subjective ratings of fattiness in food are positively correlated with the strength of neural responses in the ventral striatum [10]. Thus, dopamine and the striatum appear to contribute to preferences for energy dense foods. Recently, it was shown that differences in diet can cause simultaneous changes in striatal circuitry and food-directed behavior [11]. However, perhaps less appreciated is the growing evidence that differences in ingested foods, especially with respect to fat, can feedback on and alter striatal dopamine signaling.

Striatal dopamine signaling is regulated by several factors including dopamine production by the enzyme tyrosine hydroxylase, pre- and postsynaptic dopamine receptors, and presynaptic dopamine transporters (DATs), all of which have been implicated in obesity [12], [13]. Alterations in DAT number or function can alter the sphere of influence of released dopamine and consequently striatal function [14], [15]. Insulin, released in response to ingested food, has been shown to influence DAT function [16], [17]. Thus, the DAT is one of the likely candidates for the effects of diet.

Recently, correlations between obesity and DAT availability as well as diet-induced alterations of DAT function have been explored. Body mass index (BMI) is negatively correlated with DAT availability in the human striatum [18]. DAT binding, and hence availability, is reduced in high fat diet (HFD) fed mice [19]. HFD -induced obesity (DIO) is associated with a reduced rate of dopamine reuptake by the DAT in rats [20]. Taken together, these studies suggest that obesity established by HFD consumption can potently influence critical presynaptic regulators of dopamine signaling – especially the DAT. However, the time course of diet-induced alterations in dopamine signaling and whether the development of DIO is requisite for changes to manifest remains unknown. We assayed DAT function by evoking dopamine release in the ventral striatum and quantifying its rate of reuptake in rats using fast-scan cyclic voltammetry. To determine if decreased dopamine reuptake was caused by reduced DAT gene expression, we measured DAT mRNA in the ventral tegmental area and substantia nigra using real-time qRT-PCR. Additionally, we used a biochemical fractionation procedure and Western blot analysis to assay striatal DAT levels in crude synaptosomal and endosomal membranes. Rats had either 2 or 6 weeks of high or low fat diet, but all measurements were made in the absence of DIO. Our results suggest that prolonged consumption of HFD, independent of DIO, decreases the rate of dopamine reuptake in the ventral striatum without decreasing DAT expression.

## Materials and Methods

### Ethics Statement

This study was carried out in strict accordance with the recommendations in the Guide for the Care and Use of Laboratory Animals of the National Institutes of Health. The protocol was approved by the Animal Care Committee at the University of Illinois, Chicago. All surgery was performed under urethane anesthesia, and all efforts were made to minimize suffering.

### Subjects

Standard male Sprague–Dawley rats (n = 67), approximately 2 months old and weighing 225–275 g upon arrival were used. Animals were individually housed in plastic cages (26.5×50×20 cm) in a temperature- (22°C) and humidity- (30%) controlled environment on a 12∶12 h light:dark cycle (lights on at 07∶00 h). Rats acclimated to the facility for one week with *ad libitum* access to standard lab chow and water.

### Food Intake and Body Weight Measurements

After acclimation, rats were weighed and randomly assigned to 1 of 4 groups that were counterbalanced for initial body weight. Two groups were maintained on low fat diet (LFD; Research Diets, New Brunswick, NJ; D12450B; 10% kilocalories from fat (3.85 kcal/g)). The other 2 groups were maintained on HFD (Research Diets; D12492; 60% kilocalories from fat (5.24 kcal/g)). For each diet, rats were maintained for either 2 or 6 weeks (wks). Thus, the 4 groups were: LFD-2 wk (n = 18), HFD-2 wk (n = 16), LFD-6 wk (n = 16) and HFD-6 wk (n = 17). All groups had *ad libitum* access to water. Food intake and body weight measurements were made three times/wk and data are reported separately for rats undergoing voltammetric recordings or DAT protein/message analysis.

### Surgical Procedures and Dopamine Measurements

Following diet exposure, a subset of rats that did not differ in body weight were prepared for voltammetric recordings (LFD-2 wk (n = 8), HFD-2 wk (n = 6), LFD-6 wk (n = 6), and HFD-6 wk (n = 7)) under urethane (1.5 g/kg) anesthesia [as in 9,21]. A guide cannula (Bioanalytical Systems, West Lafayette, IL) was positioned above the ventral striatum (1.3 mm anterior, 1.5 mm lateral from bregma), a chlorinated silver wire (Ag/AgCl) reference electrode was implanted in the contralateral cortex and both were secured to the skull with stainless steel screws and dental cement. A micromanipulator containing a carbon-fiber electrode (CFE) was inserted into the guide cannula and the electrode was lowered into the ventral striatum. The CFE and reference electrode were connected to a headstage and the potential of the CFE was scanned from −0.4 to +1.3 V (vs. Ag/AgCl) and back (400 V/s; 10 Hz). A bipolar stimulating electrode (Plastics One, Roanoke, VA) was then gradually lowered into the ventral tegmental area/substantia nigra pars compacta (VTA/SNpc; 5.2 mm posterior, 1.0 mm lateral and initially 7.0 mm ventral from bregma) in 0.2 mm increments. At each increment, a train of current pulses (60 pulses, 4 ms per pulse, 60 Hz, 400 µA) was delivered. When the stimulating electrode is positioned in the VTA/SNpc and the CFE is in the striatum, stimulation reliably evokes dopamine release - extracted from voltammetric data using principal component analysis [9], [22]; and converted into concentration after each CFE is calibrated in a flow injection system following each experiment [23]. The position of the stimulating electrode was optimized for maximal release. The CFE was then allowed to equilibrate for 10 min before starting the experiment. Dopamine release was evoked by electrical stimulation of the VTA/SNpc (same parameters as above), and the resultant changes in dopamine concentration were calculated from −5 s to 10 s relative to stimulation. Immediately following stimulation, rats were injected with cocaine hydrochloride dissolved in 0.9% saline (10 mg/kg i.p.) and, 10 min later, the stimulation was repeated. Applied voltages, data acquisition, and analysis were performed using software written in LabVIEW (National Instruments, Austin, TX, USA) [22].

### Dopamine Reuptake

Dopamine reuptake was modeled using Demon Voltammetry Analysis Software (24; Wake Forest University, Winston-Salem NC). Here we report the decay constant tau as our measure of the rate of dopamine reuptake. Tau is derived from an exponential curve fit that encompasses the majority of the dopamine clearance curve and is highly correlated (r = .9899) with K_m_, the apparent affinity of dopamine for the DAT [24]. To determine the effect of cocaine on peak dopamine concentration we compared values obtained before and after administration (% change).

### Histology

After each recording, a stainless steel electrode (A-M Systems #571500, Sequim, WA) was lowered to the same depth as the CFE and a lesion (10 µA, 4 s) was made to mark the recording location. Brains were removed and stored in 10% formalin. Light microscopy was used to identify the lesion location on coronal sections (50 µm) through the striatum. All recordings reported here were made in the ventral striatum [25].

### Subcellular Fractionation of Striatal Tissue

Rats (LFD-2 wk, HFD-2 wk, LFD-6 wk, and HFD-6 wk; n = 10/group; no difference in body weight) were killed by decapitation. Biochemical fractionation was performed using the protocol described in [26], with minor modifications. Brains were rapidly removed, frozen in isopentane and sliced on a cryostat (HM505E, Microm, Walldorf, Germany, −20°C) until reaching the striatum. Bilateral 1-mm^3^ punches through the ventral striatum (average tissue weight: 15.2 mg) were homogenized for 20 s in 0.8 ml ice-cold TEVP (10 mM Tris base, 5 mM NaF, 1 mM Na_3_VO_4_, 1 mM EDTA, 1 mM EGTA, pH 7.4) +320 mM sucrose buffer. A 100 µl aliquot of total homogenate (H) was saved. The remainder of H was centrifuged at 800×g for 10 min at 4°C. The pellet (P1, nuclei and large debris) was resuspended in 0.2 ml TEVP buffer and saved. The supernatant (S1) was removed and placed in a clean tube on ice. S1 was centrifuged at 9200×g for 15 min at 4°C to generate a pellet (P2, crude synaptosomal membranes) and a supernatant (S2). P2 was rinsed once in TEVP +35.6 mM sucrose buffer and then resuspended in 0.25 ml of TEVP +35.6 mM sucrose buffer, vortexed gently for 3 s and hypo-osmotically lysed by keeping the sample on ice for 30 min. Supernatant (S2) was collected and spun at 165,000×g for 2 h to generate a pellet (P3, light membranes, recycling endosomes) that was resuspended in TEVP (0.1 ml) and saved. All samples were kept at −80°C until polyacrylamide gel electrophoresis.

### Gel Electrophoresis and Western Blotting

Protein content was determined using the Bio-Rad DC Protein Assay kit (Hercules, CA), and the concentration of each sample was adjusted to 0.3 mg/ml protein. NuPAGE LDS (lithium dodecyl sulfate) sample buffer (Invitrogen, Carlsbad, CA) and 50 mM dithiothreitol were added to each sample prior to heating at 70°C for 10 min. To load equivalent amounts of protein for each fraction, 3 µg of each sample were loaded into NuPAGE Novex 4–12% Bis-Tris gels (Invitrogen) for separation by gel electrophoresis. Proteins were subsequently transferred to polyvinylidene fluoride membrane (PVDF) (PerkinElmer Life Sciences, Boston, MA). Nonspecific binding sites were blocked for 2 hr at room temperature in blocking buffer (5% nonfat dry milk in PBS and 0.02% Tween 20 [PBS-T]). Blots were then incubated in primary antibody (1∶3000 mouse monoclonal anti-NR2B [#05–920, Millipore], 1∶5000 rabbit anti-DAT [#AB2231, Millipore], and 1∶1000 mouse monoclonal anti-transferrin receptor (TfR) [#13–6800, Invitrogen]. Blots were cut into 3 parts: high (>97 kDa), medium (46–97 kDa), and low (<46 kDa) weights and each part probed with an antibody that recognized a protein within that weight range. Apparent molecular weights for the antibodies used are: NR2B, 180 kDa; DAT, 75, 64, and 50 kDa; TrfR, 95 kDa. After probing medium weight range blots for DAT, antibodies were stripped by incubation with stripping buffer (62.5 mM Tris, 2% SDS, 100 mM β-mercaptoethanol, pH 6.8) for 15 min at 50°C. Blots were subsequently re-blocked and probed with anti-TfR. SeeBlue Plus 2 (Invitrogen) pre-stained standards were run for molecular weight estimation.

Protein immunoblots were analyzed using Carestream Molecular Imaging Software 5.0. Net intensity (the sum of the pixels within the band of interest minus the sum of the background pixels) was determined for each band. To permit comparisons between blots, data were normalized to the LFD controls at 2 and 6 wks. Data are expressed as the mean fold induction compared to LFD ± SEM.

### Quantitative Real-time Reverse Transcriptase Polymerase Chain Reaction (qRT-PCR)

Following collection of striatal punches for western blot analysis, frozen brains were coronally sectioned on the microtome until reaching the VTA/SN. Bilateral 1-mm^3^ punches of VTA and SN tissue (average tissue weight = 15.0 mg) were made and RNA was extracted using PureLink RNA Mini Kit (Invitrogen). RNA quality and quantity were assessed using an RNA 6000 Nano Chip (Agilent, Santa Clara, CA) on an Agilent Bioanalyzer 2100. RNA integrity number (RIN) exceeded 7 for all samples, indicating high quality. One microgram of total RNA was used to synthesize cDNA using iScript cDNA Synthesis Kit (BioRad) in a ThermoHybaid iCycler (Thermo Scientific). Primers specific for DAT (Slc6a3; Forward primer: GGAAGCTGGTCAGCCCCTGCTT, Reverse primer: GAATTGGCGCACCTCCCCTCTG), β-actin (Nba; Forward primer: AGGGAAATCGTGCGTGACAT; Reverse primer: AAGGAAGGCTGGAAGAGAGC), and TATA box binding protein (Tbp; Forward primer: ACCTAAAGACCATTGCACTTCGTGCC; Reverse primer: GCTCCTGTGCACACCATTTTCCC) genes (Genbank accession numbers NM_012694, NM_031144, and NM_001004198) were designed using NCBI Primer-BLAST (http://www.ncbi.nlm.nih.gov/tools/primer-blast/) and purchased from Integrated DNA Technologies (Coralville, Iowa). Melt curve analysis and polyacrylamide gel electrophoresis confirmed the specificity of the primers. The DAT, β-actin, and Tbp amplicons are 266, 182, and 136 base pairs in length, respectively.

A Q-PCR kit (iQ SybrGreen Supermix, BioRad) was used. The reaction was carried out on a MyiQ Single Color Real-Time PCR Detection System (BioRad) in a volume of 20 µl, with 2 µL of 3 µM forward and reverse primers and 4 µL cDNA sample diluted 1∶10. PCR cycling conditions were 95°C for 5 min; 40 cycles at 94°C for 15 s, 60° for 15 s, 72°C for 15 s. Data were collected at a read temperature of 84°C for 15 s based on the amplicon melt temperatures. Standard dilution curves were generated for each primer set by serially diluting (1.00, 0.2, 0.04, and 0.008-fold) a master cDNA stock comprising an equal mix of cDNA from all treatment groups. The log_10_ of the dilution values was plotted against the threshold cycle values for the standard curves. MyiQ Optical System Software (BioRad) was used to analyze the data. Samples containing no cDNA template and samples from cDNA reactions containing no reverse transcriptase were run as controls for contamination and amplification of genomic DNA, respectively. Reported values were normalized to the average values of the internal standards ß-actin and Tbp for each sample. Data are expressed as mean relative levels of DAT/internal standards mRNA±SEM.

### Statistical Analyses

DAT expression dynamically changes during the lifecycle in both humans [27] and rats [28], [29]. Additionally, the dopamine and behavioral response to cocaine also changes as young rats mature [30]. Thus, measurements of DAT could co-vary with age and prohibit meaningful comparisons between the 2 wk and 6 wk groups. Therefore, group means for food intake, body weight, peak dopamine concentration, tau, % change, and relative gene expression were compared separately for 2 and 6 wk groups using Student’s unpaired t-test. For western blot analyses, group differences in normalized DAT band intensity were compared separately for 2 and 6 wk groups using two-way repeated-measures ANOVA (dietXfraction). All statistical analyses were performed in Graph Pad 5 (Prism Inc.).

## Results

### HFD Promotes Increased Fat Consumption

**Figure 1:**
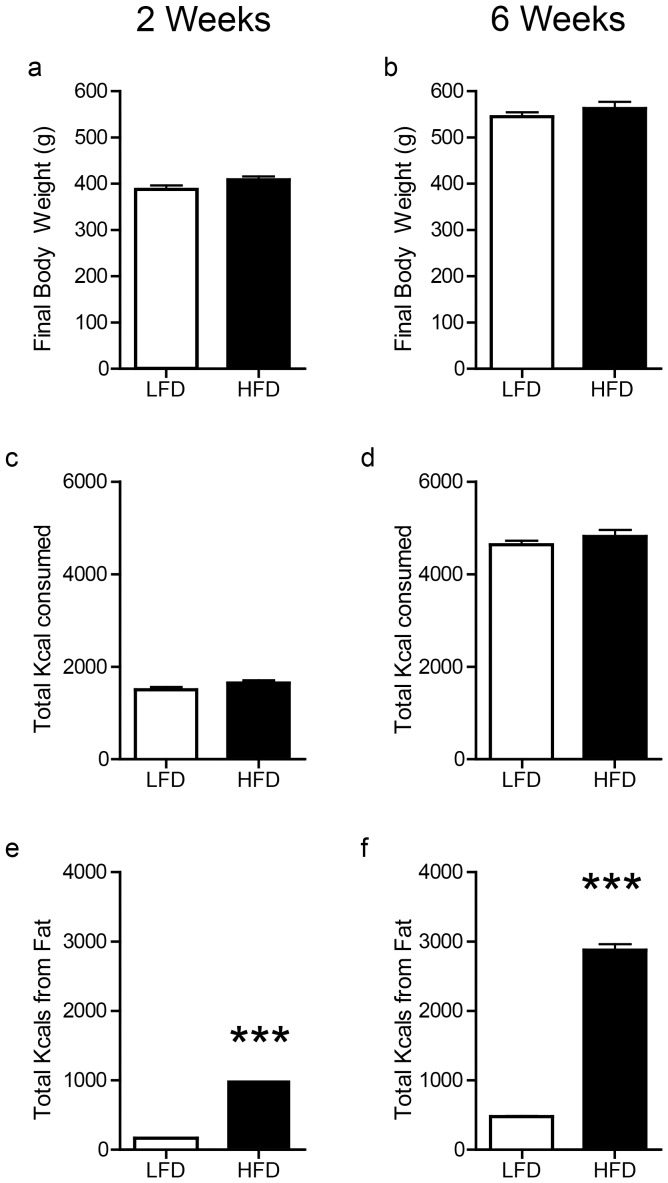
Food intake and body weight measurements. There were no differences between HFD and LFD in final body weight (**a–b**) or total kilocalories consumed (**c–d**) following either 2 or 6 weeks of diet exposure. (**e–f**) HFD rats consumed significantly more kilocalories from fat than LFD rats in both 2 week and 6 weeks conditions (***p<0.001).

Prior to the onset of diet exposure there were no differences in initial body weight in the 2 wk (LFD: 275.22+/−4.1 g; HFD: 280.87+/−4.8 g; *p* = 0.37), or 6 wk (LFD: 287.31+/−4.9 g; HFD: 289.44+/−5.1 g; 6 wk *p* = 0.97) groups. Despite consuming diets of drastically different composition, we found no differences in body weight between diet groups following either 2 or 6 wks (Fig. 1a–b; both n.s.). There was also no difference in the total kcals consumed between groups following both 2 and 6 wks of diet exposure (Fig. 1c–d; n.s.). However, HFD rats consumed significantly more kcals from fat (Fig. 1e–f; 2 wks: t(32) = 25.59; 6 wks: t(31) = 27.54; *p*<0.0001 for both diet durations).

### Prolonged HFD Reduces the Rate of DA Reuptake

**Figure 2:**
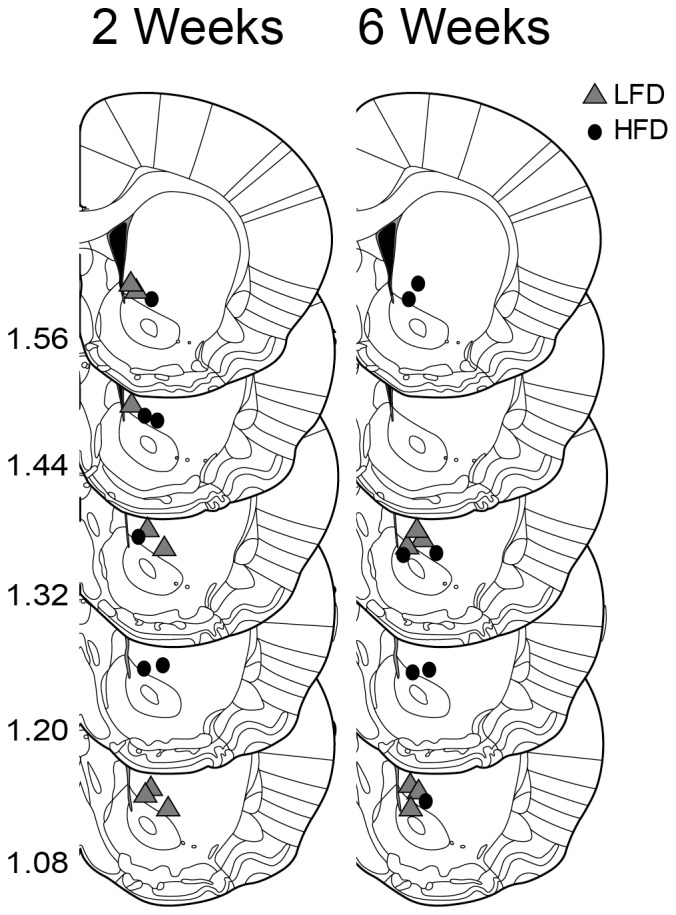
Histological verification of recording sites for reuptake analysis. Recordings sites for LFD fed rats are coded by gray triangles and for HFD rats by black circles. Numbers indicate distance in mm anterior to Bregma. Figure adapted from Paxinos and Watson 2006.

**Figure 3:**
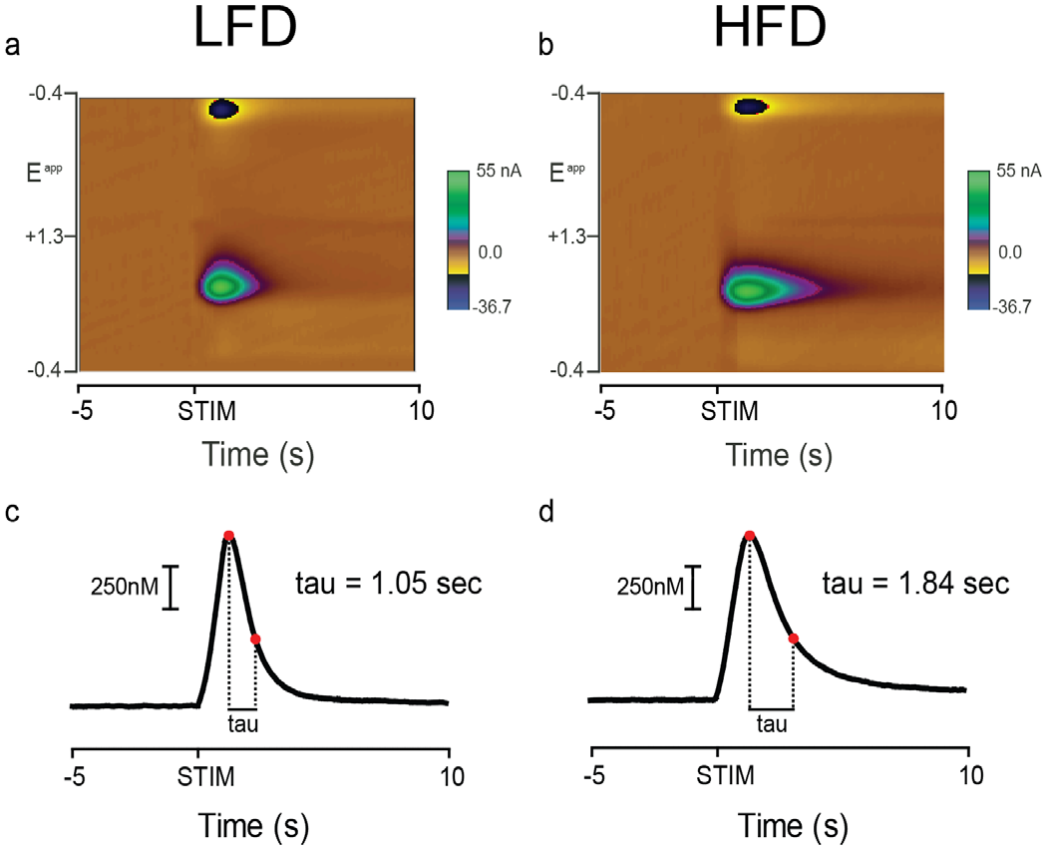
Electrical stimulation of the VTA/SNc evokes a phasic spike in dopamine concentration. Representative examples of data acquired after 6 weeks of diet exposure. **a)** Background-subtracted color plot shows current changes at different potentials of the electrode before (−5 to 0 s relative to onset) and after (0.1 to 10 s relative to onset) electrical stimulation (STIM) of the VTA/SNc. Time is the abscissa, the electrode potential is the ordinate, and current changes are encoded in false color. Dopamine [identified by its oxidation (+0.6 V; green) and reduction (−0.2 V; blue) features] transiently increased in response to the stimulation in this LFD-6 wk rat. **b)** Same as in a), except from a HFD-6 wk rat. **c)** Dopamine concentration as a function of time is extracted from the color plot in a) and tau is identified via curve fit. Two red dots mark the peak and the dopamine concentration at the time point when tau is reached. Tau is indicated at right. **d)** Same as in c) but data is extracted from b).

**Figure 4:**
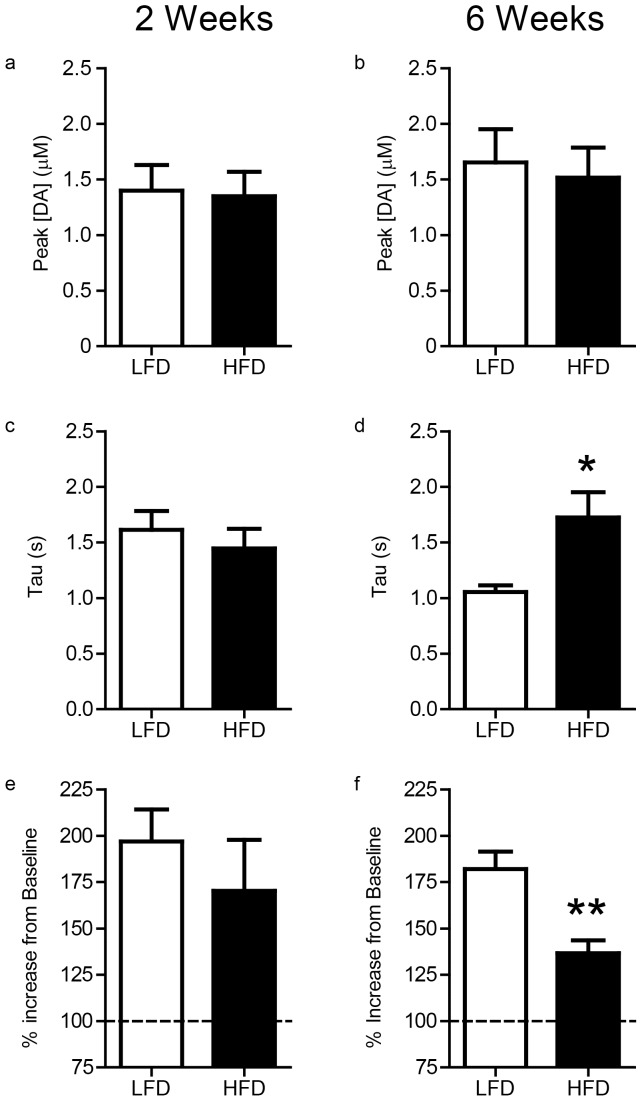
Six weeks of high fat diet reduces the rate of dopamine reuptake and attenuates the dopamine response to cocaine. Average peak dopamine concentration evoked by VTA/SNpc stimulation following either 2 (**a**) or 6 weeks (**b**) of diet exposure before cocaine injection. **c–d**) Average Tau following 2 (**c**) wks or 6 wks (**d**) of diet exposure. Tau was significantly greater for HFD-6 wk rats relative to LFD-6 wk rats (*p<0.05). **e–f**) Percent change in peak evoked dopamine concentration after cocaine injection for 2 (**e**) and 6 (**f**) weeks of diet exposure. Percent change was significantly smaller in HFD-6 wk relative to LFD-6 wk rats (**p<0.01).

Voltammetric recordings were made in the ventral striatum (Figure 2). Figure 3 shows representative electrically evoked changes in dopamine concentration acquired from rats following 6 wks of diet. At baseline, the magnitude of evoked dopamine did not differ between diet groups and across diet durations (Fig. 4a–b, both n.s). However, inspection of individual examples suggested the rate of decay following peak dopamine concentration differed between diet groups after 6 wks of diet exposure (Figure 3 a–b for examples). The rate of decay is due primarily to dopamine clearance by the DAT [31], which we modeled as a single phase exponential to determine tau. There were no differences between diet groups following 2 wks of diet exposure (Fig. 4c). However, after 6 wks of diet exposure, tau was significantly greater in HFD-6 wk rats relative to LFD-6 wk (Fig. 4d; t(11) = 2.668; *p*<0.05). Thus, 6 wks of HFD reduces the rate of dopamine clearance in the ventral striatum compared to animals that consumed a LFD.

### Prolonged HFD Decreases the DA Response to Cocaine

To further probe for diet-induced alterations in DAT, we injected rats with the DAT blocker cocaine. Peak dopamine concentration following electrical stimulation is caused by dopamine release but is also limited by simultaneous removal of dopamine by the DAT [21]. We characterized the effect of cocaine on dopamine transmission by calculating the change in the magnitude of evoked dopamine relative to pre-drug values (%change). Two wks of HFD did not affect %change relative to LFD (Fig. 4e; n.s.). However, following 6 wks of diet exposure, %change was significantly blunted in HFD relative to LFD (Fig. 4f; t(10) = 4.014; *p*<0.01). Our results suggest that 6, but not 2 wks, of HFD exposure reduces the dopamine response to cocaine.

### Prolonged HFD Exposure Reduces DAT Protein Expression in Synaptosomal Membranes

**Figure 5:**
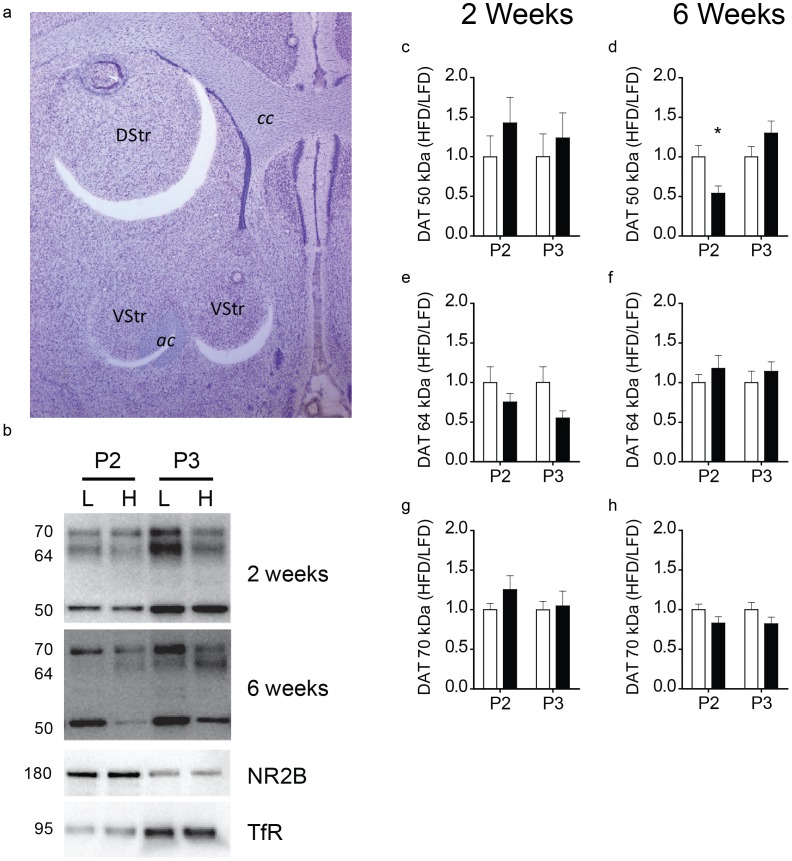
Consumption of a high-fat diet decreases membrane associated DAT protein in the ventral striatum. **a)** Representative image showing the (2) 1×1 mm tissue punches taken from the ventral striatum that were combined for DAT protein analysis. VStr = Ventral Striatum; DStr = Dorsal Striatum; cc = corpus callosum; ac = anterior commissure. **b)** Representative western blots of the data presented in c–h. L = LFD; H = HFD; TfR = transferrin receptor; NR2B = NR2B subunit of NMDA receptor. **c)** There were no differences in 50 kD DAT protein for either P2 or P3 fractions following 2 weeks of diet exposure. **d)** 50 kD DAT protein is significantly reduced in the P2 (* = *p*<.05), but not P3 fraction of ventral striatal tissue in HFD-6 wk relative to LFD-6 wk rats. There were no differences in 64 kD DAT protein following either 2 (**e**) or 6 weeks (**f**) of diet exposure. There were no differences in 70 kD DAT protein following either 2 (**g**) or 6 weeks (**h**) of diet exposure.

To determine if effects of prolonged HFD were due to changes in DAT number, DAT protein levels were quantified in total tissue homogenates (H fraction), synaptosomal membranes (P2 fraction) and intracellular recycling endosomes (P3 fraction). DAT is an *N*-linked glycoprotein with an apparent molecular weight of between 50 and 80 kDa due to increasing levels of glycosylation as the protein matures [32]. Fractionation was confirmed by enriched expression of the NR2B subunit of the NMDA receptor in synaptosomal membrane fraction and of the transferrin receptor in the endosomal fraction (for example blot see Fig. 5b). We found no differences in total DAT protein after 2 and 6 wks of diet exposure (data not shown). To test for fraction-specific differences in DAT protein, we used two-way repeated-measures ANOVA (dietXfraction). Consistent with the voltammetry experiments, 2 wks of diet exposure was insufficient to alter levels of any of the DAT isoforms in either P2 or P3 fractions (Fig. 5. c,e,g; all n.s.). However, following 6 wks of diet exposure, there was a significant dietXfraction interaction (*F*_(1,18)_ = 8.361, *p*<0.01); Fig. 5d) for the 50 kD isoform of the DAT. Thus, prolonged HFD significantly reduced the 50 kD isoform of the DAT in the P2 fraction and caused a trend towards an increase in the P3 fraction. We found no effect of diet or fraction on either the 64 kD (Fig. 5f; n.s.) or the 70 kD (Fig. 5h; n.s.) DAT isoforms.

**Figure 6:**
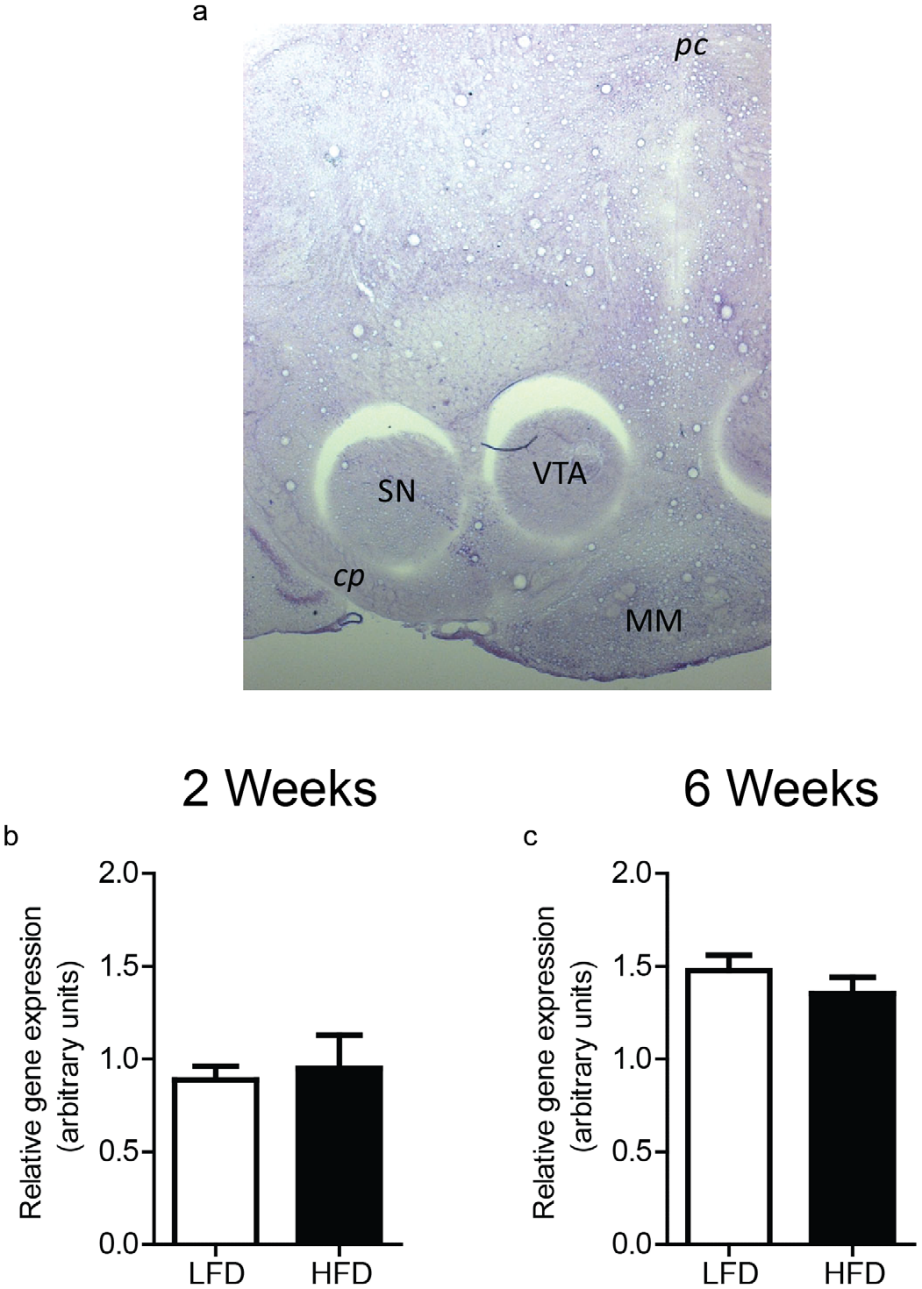
High-fat diet consumption does not alter DAT mRNA levels. a) Representative image showing 1×1 mm tissue punches taken from the VTA/SN and combined for DAT mRNA analysis. cp = cerebral penduncle; pc = posterior commissure; MM = medial mammillary nucleus. There were no differences in relative DAT mRNA levels following either 2 weeks (**b**) or 6 weeks of diet exposure (**c**).

To determine if decreased levels of DAT protein in the P2 fraction was due, in part, to a reduction in DAT transcription, VTA/SNc DAT mRNA levels were measured in the same rats as above (Fig. 6a for example). We observed no differences between diet groups in midbrain DAT mRNA after either 2 or 6 wks of diet exposure (Fig. 6b–c; both n.s.). Thus, differences in DAT protein levels within the ventral striatum are unlikely to be due to deficits in DAT production.

## Discussion

Prolonged HFD consumption can lead to DIO and plasticity within the central nervous system. Dopamine neurons and striatal dopamine receptors appear to be one set of CNS targets that are affected by a HFD and in obese individuals [11], [13], [33]. Here we report that a HFD reduced the rate of dopamine reuptake in the ventral striatum and this effect was dependent on the duration of exposure. Importantly, the effect of HFD on DAT function occurred in the absence of DIO. While we did not directly measure markers of body adiposity in this study, animals have been traditionally classified as DIO or diet-resistant based on solely on body weight gain following exposure to a HFD [34]. Prolonged HFD significantly attenuated the ability of cocaine, which interferes with the DAT, to potentiate the magnitude of dopamine release. We quantified DAT protein levels in the ventral striatum using Western blot analysis – distinguishing between DAT localized within subcellular fractions enriched for either the plasma membrane or recycling endosomes. We found a significant reduction in an immature isoform of the DAT associated with the plasma membrane. Thus, prolonged HFD appears to reduce the rate of dopamine reuptake via the DAT likely by interfering with DAT trafficking or perhaps maturation but not by decreasing DAT gene expression or DAT mRNA stability. Moreover, a period between two and six weeks of exposure to a HFD appears to be the earliest tipping point for diet-induced plasticity with respect to the DAT.

Obesity is correlated with multiple aspects of striatal dopamine signaling, including DAT availability in both humans [18] and mice [19]. However, only recently was it shown that the development of DIO alters the rate of dopamine reuptake in rats [20]. While this study demonstrated impaired dopamine reuptake following exogenously applied dopamine after only 4 weeks of HFD, the animals that were maintained on a HFD were selected based on initial weight gain and thus could represent a unique population. Consistent with this view, HFD animals continued to eat more calories and gain more weight compared to LFD controls. Another recent study reported impaired dopamine reuptake following 12 weeks of HFD in out-bred rats [35]. However, there were significant differences in body weight between the animals fed a HFD versus a standard lab chow diet when the reuptake measurements were made. Therefore, it remained unclear whether impairments in dopamine reuptake emerge as a direct result of, or precede, DIO development. In contrast to these recent reports, we found no differences in body weight or total kcal consumption between our diet groups when reuptake measurements were made. That we found differences in dopamine reuptake after 6, but not 2, weeks of HFD suggests that diet-induced alterations in dopamine reuptake are a response to chronic, but not acute, changes in diet composition. Additionally, our results suggest that instead of being a result of obesity, diet-induced alterations in DAT could contribute to the development of the disease. Future studies will need to address whether or not animal populations that are differentially susceptible to DIO [34] have preexisting differences in DAT expression/function or are differentially susceptible to diet-induced changes in DAT.

To our knowledge, this is the first study demonstrating that a HFD reduces the dopamine response to cocaine. Given dopamine’s role in drug reward, our results are consistent with previous work demonstrating that rats fed a HFD for approximately 6 weeks are slower to acquire cocaine self-administration than animals fed a control diet [36]. Importantly, this effect was also independent of DIO development. Additionally, rats selectively bred for susceptibility to DIO show reduced cocaine place preference, suggesting that the rewarding properties of cocaine are blunted in these animals [37]. The decreased response to cocaine we observed in HFD-6 wk rats could be due to reduced striatal DAT availability. However, cocaine also increases dopamine signaling through non-DAT dependent mechanisms. Specifically, HFD could have impaired cocaine-induced mobilization of reserve dopamine vesicles [38]. Cocaine also attenuates GABA transmission onto dopamine neurons within the VTA [39] and induces oscillations in the firing rate of dopamine cell bodies [40]. Any or all of these processes could also have been affected by a HFD. Future research will need to address the mechanisms underlying how a HFD modifies the rewarding aspects of cocaine and/or the potential for drug-induced neural adaptations [18]. Consumption of a HFD attenuates both the behavioral [41] and dopamine response [20], [42] to amphetamine, which also interferes with the DAT. Importantly, rats whose intake of a HFD was calorically matched to that of rats fed a control diet do not develop DIO but still fail to develop an amphetamine conditioned place preference [41]. Together with the data presented here, it appears that consumption of a HFD blunts the response to psychostimulants. All drugs of abuse influence the dopamine system, and drug-induced enhancement of dopamine signaling is thought to be critical for the development of addiction [43]. Thus, the reduced response to cocaine in HFD rats is consistent with reports that obese humans have a significantly lower lifetime risk of developing a substance abuse disorder [44]. Future work will need to address whether the subjective rating of cocaine reward differs in obese individuals compared to normal weight controls.

Our western blot analysis suggests that prolonged consumption of a HFD does not affect total striatal DAT protein, but instead reduces the integration of the non-glycosylated 50 kDa DAT isoform into synaptosomal membranes. While DAT glycosylation improves the rate of dopamine transport and increases membrane surface stability [45], [46], [47], non-glycosylated DAT from humans [45], [46] as well as rats [47] readily transports dopamine. Additionally, immunolabeling experiments reveal that levels of non-glycosylated DAT are higher in the ventral compared to dorsal striatum in both monkeys and humans [47]. Taken together, these studies suggest that the decreased membrane levels of 50 kDa DAT could contribute to the reuptake deficit we observed in 6 wk HFD rats. Our data are consistent with a previous study showing HFD consumption reduces DAT availability in the ventral striatum of mice [19]. However, this study did not measure DAT localization in different intracellular compartments. Additionally, our findings are consistent with a study showing reductions in cell surface DAT in the striatum of DIO rats [20]. This study also reported that total DAT protein levels were unaffected by diet in the DIO model. We expand this finding to show that total DAT protein is also unaffected by a HFD in out-bred rats. Therefore, prolonged consumption of a HFD does not alter DAT expression, but may interfere with DAT trafficking or maturation.

The lack of differences in VTA/SNpc DAT mRNA levels after either 2 or 6 wks of HFD exposure further supports the notion that overall DAT levels were unaffected by our diet manipulations. This result contrasts with a previous report showing reduced DAT mRNA in the mouse VTA following 17 weeks of HFD consumption [12]. However, in this study DAT mRNA levels were measured after the diet groups had differed in body weight for 12 weeks. Thus, their results likely represent late stage adaptations to DIO. In summary, our data provides strong evidence that exposure to a HFD leads to functional changes in striatal dopamine reuptake by decreasing membrane-associated DATs without altering total DAT expression. Importantly, we report that diet-induced disruptions in the DAT can occur prior to the onset of DIO, suggesting that these alterations could contribute to the development of obesity.

Our data add to a growing literature implicating diet in the regulation of dopamine function, and provide further evidence that diet induced changes in DAT expression leads to functionally relevant changes in dopamine signaling. Diet-induced alterations in the dynamics of striatal dopamine signaling via the DAT are likely to have consequences for feeding behavior. Food-related stimuli evoke phasic increases in striatal dopamine [9], [48], [49], which likely reinforce and strengthen food-directed actions [50]. Here we show that 6 weeks of HFD consumption prolongs the duration of phasic dopamine release by decreasing membrane associated DATs in a region of the striatum where dopamine function is essential for food intake [51]. Diet-dependent alterations in DAT could promote a feed-forward mechanism whereby prolonged dopamine signals evoked by food stimuli increase activation of low affinity striatal dopamine D1 receptors, which are critical for approach behaviors [52], [53], [54]. Over time, prolonged elevation of striatal dopamine could promote adaptations, such as downregulation of dopamine D2 receptors (D2R), which has been demonstrated in both human and rodent models of obesity [11], [33]. Our study suggests that the development of obesity is not a requisite to alter dopamine reuptake. Thus, diet-related decreases in membrane DAT could precede and contribute to the onset of D2R downregulation, obesity, and compulsive eating behavior that develops over the course of HFD consumption [11].
